# Influence of ageing on the microarchitecture of the spleen and lymph nodes

**DOI:** 10.1007/s10522-017-9707-7

**Published:** 2017-05-13

**Authors:** Vivian M. Turner, Neil A. Mabbott

**Affiliations:** The Roslin Institute and Royal (Dick) School of Veterinary Sciences, University of Edinburgh, Easter Bush Campus, Roslin, Midlothian, EH25 9RG UK

**Keywords:** Ageing, Spleen and lymph nodes, Marginal zone, Follicular dendritic cells, Macrophages, Microarchitecture

## Abstract

The elderly have a decreased response to vaccination and an increased susceptibility to infectious diseases. The spleen and lymph nodes are important secondary lymphoid organs where immune cells can rapidly respond to pathogenic material in the blood and lymph in order to mount long-term adaptive immune responses to those pathogens. In aged mice and humans structural changes occur to both the spleen and lymph nodes. These structural changes affect the functioning of the immune cells within, which may ultimate result in less effective or decreased immune responses. This review describes our current understanding of the structural changes that occur to the spleen and lymph nodes of elderly mice. However, where data are available, we also discuss whether similar changes occur in tissues from elderly humans. A particular focus is made on how these structural changes are considered to impact on the functioning of the immune cells within. The world’s population is currently living longer than ever before. The increased incidence and severity of infectious diseases in the elderly has the potential to have a significant impact on the health care system if solutions are not identified. A thorough understanding of the molecular causes of these ageing-related structural changes to the spleen and lymph nodes may help to identify novel treatments that could repair them, and in doing so, improve immune responses and vaccine efficacy in the elderly.

## Introduction

The immune system is severely compromised in the elderly. As a consequence of this immunosenescence elderly humans exhibit a decreased ability to respond to vaccination (Osterholm et al. 2012; Bondada et al. 2000), have increased susceptibility to viral and bacterial infections (Quandelacy et al. 2014; van der Poll and Opal 2009) and increased incidence of cancer and autoimmune diseases. The spleen and lymph nodes are structurally organised to ensure the efficient uptake, processing and responses to pathogenic material present in the blood and lymphatic fluid (Junt et al. 2008). In the spleen it is the marginal zone region, consisting of specialised populations of macrophages, B cells and stromal cells, which is involved in the initial response to blood-borne pathogens (Junt et al. 2008). In the lymph node lymphatic fluid enters into the subcapsular region, and it is the subcapsular sinus macrophages and stromal cells within it which are initial responders (Junt et al. 2008). In either situation these early responding cell populations act to initiate an efficient immune response to pathogenic material to mediate the fast recovery of the host from infection. Many studies have addressed the influence of ageing on the thymus and T cell responses (for recent review see: (Akbar et al. 2016). Ageing-related changes to the structural organisation of spleen and lymph nodes have also been described in humans and animals. This review discusses our current understanding of these age-related changes to the structure of spleen and lymph nodes, and how they are considered to impact on immune function. Although much of our understanding has been derived from the analysis of aged animals, especially mice, where data were available we also discuss the changes which have been observed tissues from elderly humans.

## Spleen

### Murine spleen structure and function

**Fig. 1:**
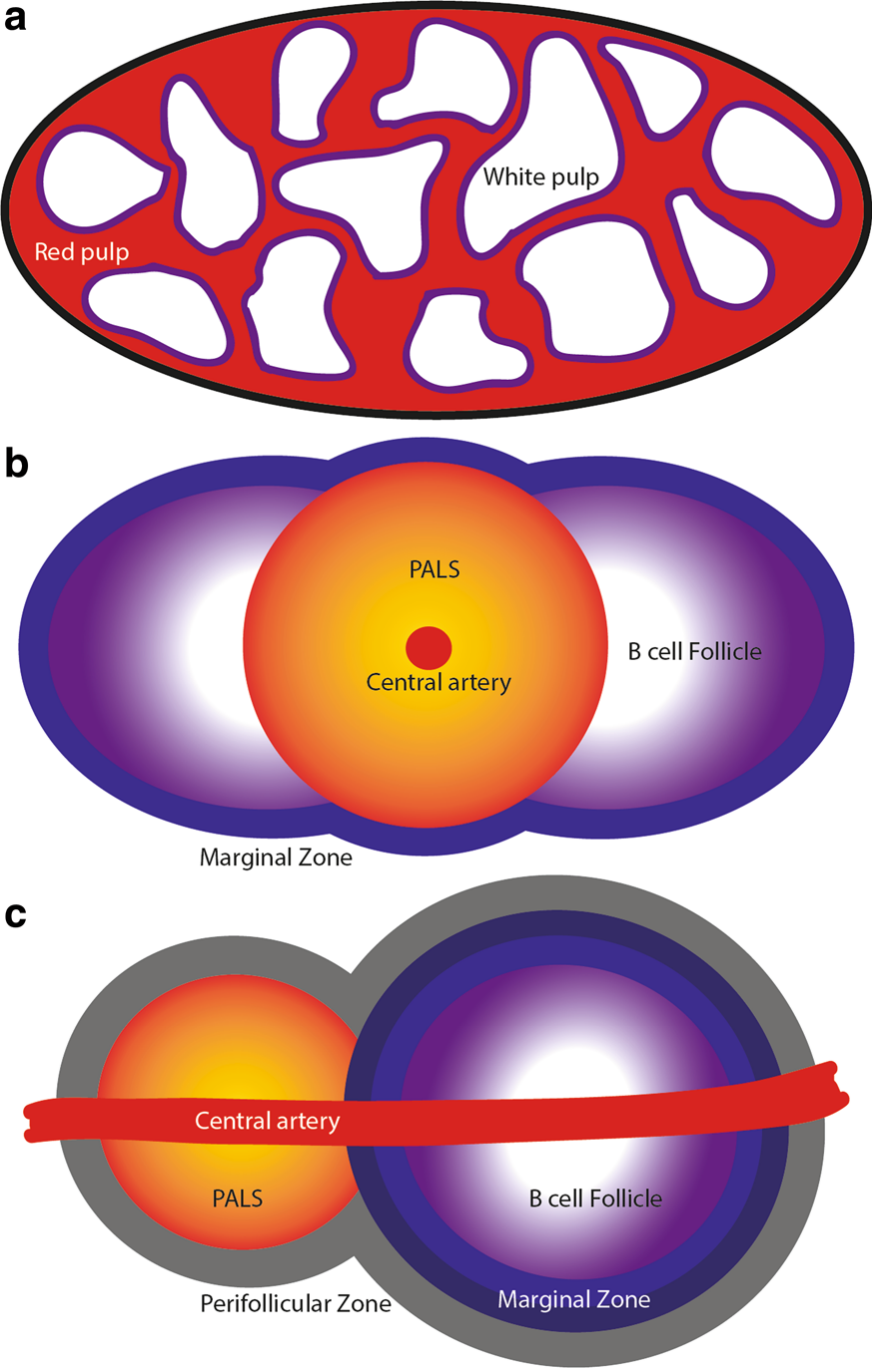
Structural organisation of the spleen. a The spleen is composed of red pulp and white pulp and is encapsulated. **b** Illustration of the mouse white pulp. The periarteriolar lymphoid sheath (PALS) in the T cell zone surrounds the central artery. Next to the PALS, B cell follicles are seen and the follicle is surrounded by the marginal zone. **c** Illustration of the human white pulp. The PALS T cell zone is adjacent to the B cell follicle. The marginal zone region surrounds the follicle and has two main layers. Surrounding the PALS and follicle is the perifollicular zone. A central artery runs through the middle of the white pulp

The spleen is composed of red pulp and white pulp regions, and is surrounded by a fibrous capsule (Fig. 1a). The red pulp contains stromal cells, important in the structure of the spleen, and red pulp macrophages, important in the clearance of pathogenic material and ageing erythrocytes (Bratosin et al. 1998). Plasmablasts and plasma cells also reside here and ensure the efficient secretion of antibodies into the blood stream (Toellner et al. 1996).

In the mouse, the white pulp is organised into lymphoid follicles (Fig. 1a). T cells reside in the periarteriolar lymphoid sheath (PALS) that surrounds the central arteriole (Mebius and Kraal 2005). Fibroblastic reticular cells (FRC), conventional dendritic cells and macrophages are also situated within the PALS. Adjacent to the PALS are the B cell follicles, which contain follicular B cells localised around stromal-derived follicular dendritic cells (FDC). The localisation of certain cell populations to the PALS and B cell follicles is regulated by the expression of homeostatic cytokines and chemokines. For example, the chemokine CXCL13 is expressed by FDC and follicular stromal cells and coordinates the migration of CXCR5-expressing (the receptor for CXCL13) B cells into the B cell follicles (Forster et al. 1996), whereas stromal cells in the PALS express the chemokine CCL21 which attracts CCR7-expressing T cells and conventional dendritic cells (Forster et al. 1999). The localisation of the T and B cell zones adjacent to each other enables efficient interactions between these two key immune cell populations during the generation of antigen-specific immune responses.

A varied repertoire of B cells with different antigen-specificities is important to enable the host to produce antibodies against a wide range of potential pathogens. To facilitate this diversity rearrangements are made in the immunoglobulin gene to produce a wide range of B cells expressing uniquely recombined immunoglobulin genes which encode antibodies with different antigen specificities. The B cells which produce high affinity antibodies are then selected by a process termed affinity maturation, which occurs within the germinal centres of lymphoid tissues. Antigen-activated B-cells migrate to the edge of the B cell follicle where they interact with T cells to receive proliferation and survival signals, forming germinal centres. Within the germinal centre the B cells proliferate and undergo a process termed hypermutation during which they accumulate further mutations in their immunoglobulin genes in order to attempt to improve the antigen-binding affinity of the antibody. These newly generated antibodies are then expressed on the B cell surface. Those B cells which receive the appropriate rescue signals, undergo further rounds of mutation and selection, before finally maturing into an antibody secreting plasma cell or memory B cell. The entirety of the germinal centre response is more thoroughly reviewed elsewhere (Sage and Sharpe 2015; Tas et al. 2016; Corcoran and Tarlinton 2016). The effects of ageing on human B cells have also been extensively covered elsewhere (Dunn-Walters 2016).

The FDC are an important stromal cell population within the B cell follicles and germinal centres. Their large surface area enables them to efficiently trap and retain large quantities of native antigen in the form of immune complexes, comprising antigen, antibody, and/or opsonising complement components. Due to their extended longevity, native antigens can be retained by FDC for months to years (Mandel et al. 1980; Heesters et al. 2013). Activated B cells interact with the antigens retained on the FDC surface. Indeed, these close interactions between FDC and B cells within the follicles are important for the B cells to receive survival signals and for the induction of antibody hypermutation and maintenance of B cell memory (Kosco-Vilbois 2003).

The splenic marginal zone is a specialised microenvironment that plays an important role in the capture and clearance of blood-borne pathogens and antigens. The marginal zone surrounds the white pulp and contains a channel of stromal cells with associated specialised populations of macrophages and B cells (Mebius and Kraal 2005). Marginal metallophilic macrophages form a layer on the outer edge of the follicle. These cells express SIGLEC1 (sialic-acid-binding immunoglobulin-like lectin 1; also known as sialoadhesin), which binds to highly sialylated glycans, terminal capping structures such as those displayed on the lipopolysaccharide of pathogenic bacteria such as *Neisseria meningitidis* (Munday et al. 1999; Jones et al. 2003). MAdCAM-1^+^ stromal cells line the sinus between the follicle and the marginal zone and provide a channel through which the blood flows as it enters the spleen. Marginal zone macrophages and marginal zone B cells are localised within the marginal zone itself. Marginal zone macrophages characteristically express the receptor SIGNR1 (specific intracellular adhesion molecule-grabbing non-integrin receptor 1), which mediates the uptake of dextran and capsular pneumococcal polysaccharides (Kang et al. 2003, 2004; Geijtenbeek et al. 2002). Marginal zone B cells are situated on the exterior of the marginal sinus. These specialised non-recirculatory B cells express B cell receptors specific for microbial polysaccharides, Toll-like receptors (TLR), complement receptors (CD21/CD35; CR2/CR1) and can self-renew. These features and their marginal zone positioning enable them to trap and concentrate blood-borne antigens on their surfaces and rapidly mount type-2 T cell-independent antibody responses to polysaccharide antigens such as those on encapsulated bacteria. Marginal zone B cells also capture blood-borne immune complexes in a complement receptor-dependent manner and rapidly shuttle them to FDC in the B cell follicles (Arnon et al. 2013; Balazs et al. 2002; Cinamon et al. 2008).

### Changes to the murine marginal zone structure and function with age

**Fig. 2:**
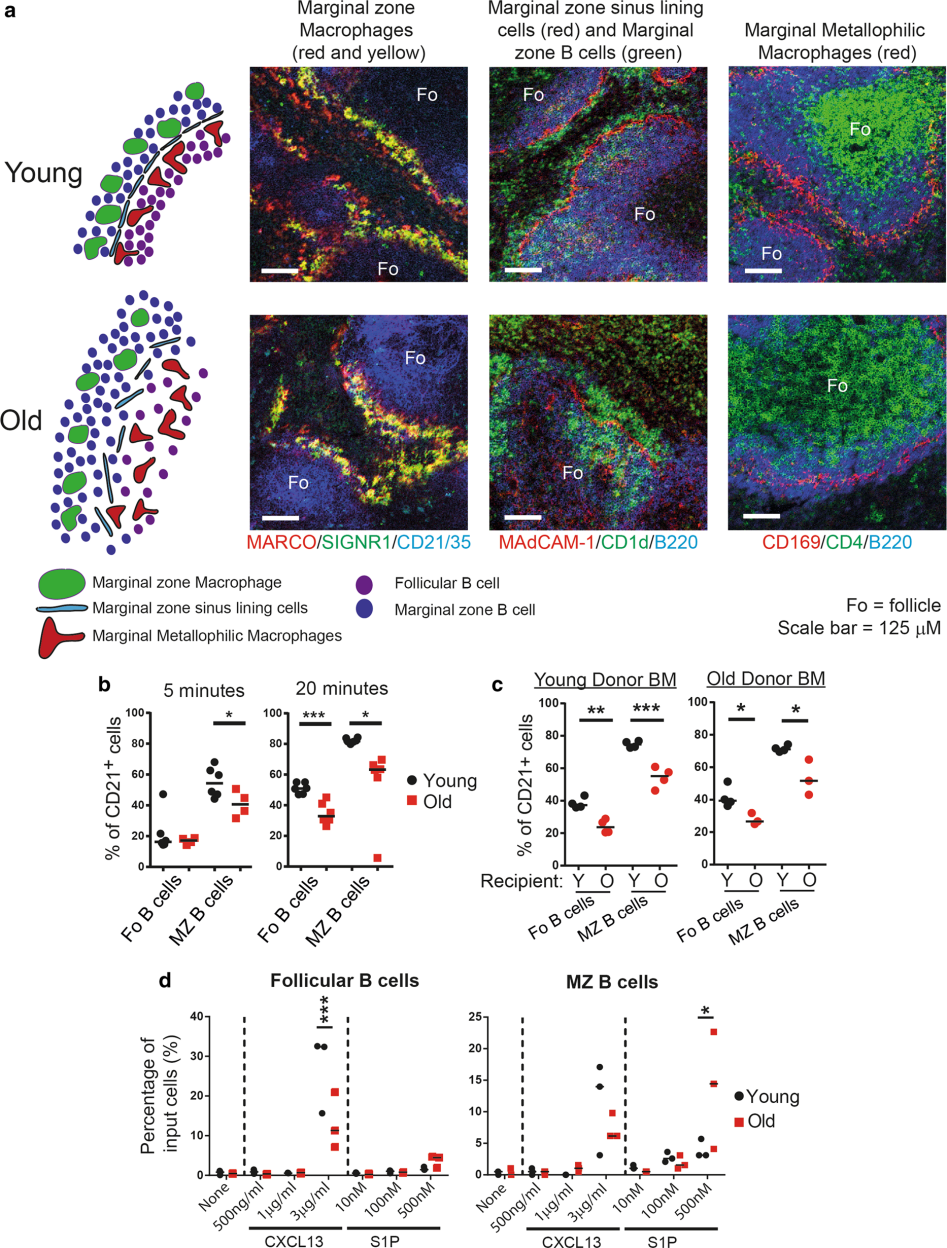
Age-related changes to the murine marginal zone. **a** In young mice the marginal zone is neatly organised. Marginal metallophilic macrophages line the edge of the B cell follicle. MAdCAM-1^+^ stromal cells mark the marginal zone sinus between the follicle and the marginal zone. In the marginal zone itself marginal zone macrophages and marginal zone B cells are intermingled and form a consistent layer around the follicle. In aged mice the region undergoes several structural changes. Both marginal zone macrophages and marginal metallophilic macrophages become disrupted in their localisation, increasing in depth and no longer forming a smooth layer. Marginal zone B cells also exhibit disrupted localisation and organisation. The MAdCAM-1^+^ stromal cell layer no longer forms a smooth, continuous barrier, becoming thicker and disjointed. Images were acquired by confocal microscopy and are representative of spleens from 2 months (young) and 18 months (old) old C57BL/6 mice. **b** Follicular and marginal zone B cells have decreased uptake of anti-CD21/35-PE in aged mice. **c** Regardless of the age of the donor bone marrow, follicular and marginal zone B cells displayed decreased uptake of anti-CD21/35-PE in aged recipients. **d** Data from in vitro chemotaxis assays show that aged follicular B cells have decreased chemotaxis towards CXCL13, whereas aged marginal zone B cells have increased chemotaxis towards sphingosine 1-phosphate (S1P). Features of this figure are reproduced from (Turner and Mabbott 2017a) under the terms of the CC-BY-NC Creative Commons Attribution Licence 4.0 (Turner and Mabbott 2017a). *BM* bone marrow, *MZ* marginal zone

The marginal zone region of the murine spleen undergoes significant structural disorganisation with age. Marginal zone macrophages have altered distribution, no longer forming a continuous boundary along the marginal zone (Fig. 2a) (Birjandi et al. 2011; Brown et al. 2012; Brown and Mabbott 2014; Aw et al. 2016; Turner and Mabbott 2017a). The MAdCAM-1^+^ marginal zone sinus lining cells in aged murine spleens also no longer form a continuous boundary between the follicle and marginal zone and become thicker in density (Fig. 2a) (Birjandi et al. 2011; Brown and Mabbott 2014; Brown et al. 2012; Turner and Mabbott 2017a). The distribution and density of the marginal metallophilic macrophages in the inner layer of the marginal zone is also disturbed in aged murine spleens (Fig. 2a) (Brown et al. 2012; Brown and Mabbott 2014; Birjandi et al. 2011; Turner and Mabbott 2017a). Functionally, marginal zone macrophages in aged BALB/c mice have been shown to have a reduced capacity to acquire dextran particles, which may be a consequence of their altered distribution and/or reduced density (Birjandi et al. 2011). However, no difference in phagocytosis per se between young and aged marginal zone macrophages was observed under in vitro conditions (Birjandi et al. 2011).

In the spleens of aged mice the distribution of marginal zone B cells is also disturbed (Brown et al. 2012; Turner and Mabbott 2017a). However, it is uncertain whether their abundance is also reduced, as the frequency of marginal zone B cells has been reported to be reduced in the spleens of BALB/c mice (Birjandi et al. 2011), but increased in the spleens of C57BL/6 mice (Miller and Cancro 2007; Turner and Mabbott 2017a). The reason for these apparent mouse background strain-dependent discrepancies is uncertain. Little is known of the consequences of the ageing-related disturbances to the splenic marginal zone to the function of marginal zone B cells within it. The disturbed microarchitecture may potentially have a significant impact on the function of marginal zone B cells, either by affecting their ability to rapidly secrete antibodies in response to blood-borne pathogens and antigens, or by impeding their ability to deliver immune complexes to FDC to initiate a germinal centre response. Marginal zone B cells are highly dynamic, constantly collecting blood-borne complement-opsonized antigens and delivering them to FDC in the B cell follicles, before migrating back to the marginal zone (Cinamon et al. 2008). We used an established in vivo antibody pulse-labelling protocol to determine whether ageing affected antigen-capture and follicular shuttling by marginal zone B cells, (Cinamon et al. 2008; Basu et al. 2011; Turner and Mabbott 2017a). Using this approach, within 5 min. after intravenous injection with phycoerytherin (PE)-conjugated anti-CD21 antibody the B cells exposed to peripheral blood (e.g.: those within the marginal zone) are labelled with the antibody, whereas those in the follicles are not. However, by 20 min post-injection a greater number of marginal zone B cells are labelled due to their migration between the follicles and marginal zone during this period. This method therefore enables the kinetics of B cell localisation and shuttling between the marginal zone and B cell follicle to be readily assessed (Cinamon et al. 2008; Basu et al. 2011). Using this in vivo assay, our data showed that the localisation and shuttling of B cells between the marginal zone and B cell follicles was significantly impaired in the aged spleen (Fig. 2b) (Turner and Mabbott 2017a).

Further analysis revealed that the follicular shuttling of marginal zone B cells derived from either young or aged bone marrow was similarly reduced in aged recipient spleens (Fig. 2c) (Turner and Mabbott 2017a). This indicated that ageing effects on splenic stromal cells were predominantly responsible for the impaired follicular shuttling of marginal zone B cells. The factors responsible for these ageing related changes to the splenic microarchitecture are uncertainn. The lysophospholipid sphingosine 1-phosphate (S1P) is abundant in the bloodstream and attracts marginal zone B cells to the marginal zone through their expression of the S1P receptors S1P_1_ and S1P_3_ (Cinamon et al. 2008). Similar to aged mice, mice lacking S1P_3_ expression have disturbed distribution of MAdCAM-1^+^ marginal zone sinus lining cells and metallophilic macrophages (Girkontaite et al. 2004), indicating that S1P-S1P_3_ signalling may also play an important role in the organisation of the marginal sinus. Whether the effects of ageing on the marginal zone microarchitecture are a consequence of reduced S1P_3_ receptor expression by stromal cells in the spleen is uncertain, but S1P levels in the blood-stream were not similar in young and aged mice (Turner and Mabbott 2017a). However, data from in vitro assays showed that migration of aged marginal zone B cells towards S1P was increased (Fig. 4d) (Turner and Mabbott 2017a) and may potentially contribute towards their enhanced retention within the marginal zone. The disturbed distribution of MAdCAM-1^+^ marginal zone sinus lining cells in the aged spleen might also physically impede the migration of B cells within this region.

Invasive pneumococcal disease from *Streptococcus pneumonia* infection is a leading cause of mortality in people >65 years old (van der Poll and Opal 2009), and the efficacy of vaccines against this disease are decreased in the elderly (Bondada et al. 2000). Since marginal zone B cell deficiencies are associated with elevated risk of pneumococcal disease and poor antibody responses to bacterial capsular polysaccharides (Timens et al. 1989; Cerutti et al. 2013), the effects of ageing on the marginal zone may likewise significantly impact on the ability of the elderly to mount effective T cell-independent antibody responses. Indeed, MZ B cell responses were disturbed in aged mice and their ability to produce antibodies in response to the T cell-independent type 1 antigen, TNP-LPS, was impaired (Turner and Mabbott 2017a).

### Changes to murine white pulp structure and function with age

De-compartmentalisation of T and B cell regions has been reported in the spleens of aged mice, with the boundaries between these regions becoming less defined as the mice age (Eaton-Bassiri et al. 2000; Aw et al. 2016). Immune responses within secondary lymphoid tissues are often associated with phases of tissue remodelling which are later resolved (Junt et al. 2008) and spleens of young mice undergo resolution following immunisation with T-independent and T-dependent antigens. However, aged spleens are unable resolve this structural disorganisation (Aw et al. 2016). Furthermore, the aged splenic microenvironment has been shown to adversely affect the migration of immature and marginal zone B cells within it (Wols et al. 2010; Turner and Mabbott 2017a). Stromal cells in the spleen play important roles in coordinating the localisation of leukocytes to specific tissue niches (El Shikh et al. 2010; Wang et al. 2011; Bonilla et al. 2012; Umemoto et al. 2012; Luther et al. 2000). The disorganised compartmentalisation of the T and B cell regions is most likely due to ageing effects on the underlying stromal cells within these zones which also become significantly altered in their distribution. For example, the FRC in the T cell zone display enlargement and expansion into B cell areas (Aw et al. 2016). The recruitment of CD4^+^ T cells to the ageing spleen is reduced and coincides with dysregulated production of homeostatic chemokines such CCL21 and CXCL13 by the stromal cells within (Lefebvre et al. 2012).

Although the distribution of CD157^+^ B cell stromal cells does not appear to be affected by ageing (Aw et al. 2016), the status of FDC networks in the B cell zone is severely compromised (Brown et al. 2012; Aydar et al. 2004; Brown et al. 2009). Aged splenic FDC are less dense and expand towards the T cell and marginal zones (Aw et al. 2016; Brown et al. 2009). This defect in FDC status has been shown to result in impaired immune-complex retention, germinal centre formation and antibody production. Germinal centres are decreased in size or absent and undergo a progressive decline in number with age (Metcalf et al. 1967; Zheng et al. 1997; Miller and Kelsoe 1995). Aged splenic FDC also display an impaired ability to trap and retain immune complexes, however, this is not due to reduced expression of complement receptors by the FDC themselves (Brown et al. 2012; Brown and Mabbott 2014; Brown et al. 2009). FDC also help to maintain the structure of the B cell follicle through their production of the B cell chemoattractant CXCL13 (Ansel et al. 2000). The expression and distribution of CXCL13 appears to be disturbed in the aged spleen, with apparent increased expression in follicular areas, but decreased expression in the vicinity of the marginal zone (Wols et al. 2010; Turner and Mabbott 2017a). The consequences of the altered CXCL13 expression to germinal centre formation and antibody responses are not known. However, aged follicular B cells have reduced migration towards CXCL13 in vitro (Fig. 2d) (Turner and Mabbott 2017a), and data suggest that aged splenic microenvironment fails to support the induction and/or maintenance of T follicular helper cells (Lefebvre et al. 2012), which may impair the subsequent generation of germinal centres.

### Human spleen structure and age-related changes

There are fundamental differences between the structures of the human and murine spleens (Mebius and Kraal 2005; Steiniger 2015). In contrast to mice, the red pulp predominates the human spleen. Within the white pulp the B cell zones are the predominant structures, with a reduced PALS and T cell zone when compared to the murine spleen (Fig. 1c). The PALS in the human spleen is also positioned adjacent to the B cell follicle (rather than surrounded by follicles as in the murine spleen) and the central arteriole runs through both the PALS and follicle (Fig. 1c). Differences are also evident in the structure of the marginal zone. In humans this region is split into two main layers with MAdCAM-1^+^ stromal cells present within each layer. The inner layer has not been shown to correlate with any lymphocyte population whilst the outer layer contains follicular B cells (Steiniger et al. 2001, 2014). Reconstructions of human spleens indicate that marginal zone is highly-vascularised implying a similar role in the filtration of pathogens and antigens from the blood (Kusumi et al. 2015). Whether marginal zone-like B cells are also present in human spleens and share similar properties to murine marginal zone B cells is currently controversial (Weill et al. 2009). Humans also have a perifollicular zone, not observed in mice, which surrounds the entirety of the PALS, the follicle and marginal zones (Fig. 1c). Sialoadhesin^+^ marginal zone macrophages accumulate around blood vessels in the perifollicular zone of the human spleen, an indicator of their potential, but as yet unstudied, role in the sampling of blood-borne antigens (Steiniger et al. 2001; Kusumi et al. 2015). Erythrocytes, granulocytes, and monocytes are also found in the perifollicular zone intermingled between additional MAdCAM-1^+^ stromal cells (Steiniger et al. 2001).

Ageing-related changes to the microarchitecture of the human spleen have been described (Alex et al. 2015). The thickness of the splenic capsule appears to be dynamically influenced by ageing, thickening post-adolescence and into middle age before thinning again in old age. Increased atrophy of splenic tissue has also been reported in individuals of advanced age (>70 years) and coincided with decreased number and size of B cell follicles with age. Although somatic hypermutation is reduced in the splenic germinal centres of aged individuals (Banerjee et al. 2002), it is uncertain whether this is direct a consequence of the structural changes. A detailed understanding of how ageing influences the microarchitecture of the human spleen is clearly required. Such data may help to identify the important factors which contribute to the failure of immune responses and vaccination in the elderly.

## Lymph nodes

### Murine lymph node structure and function

**Fig. 3:**
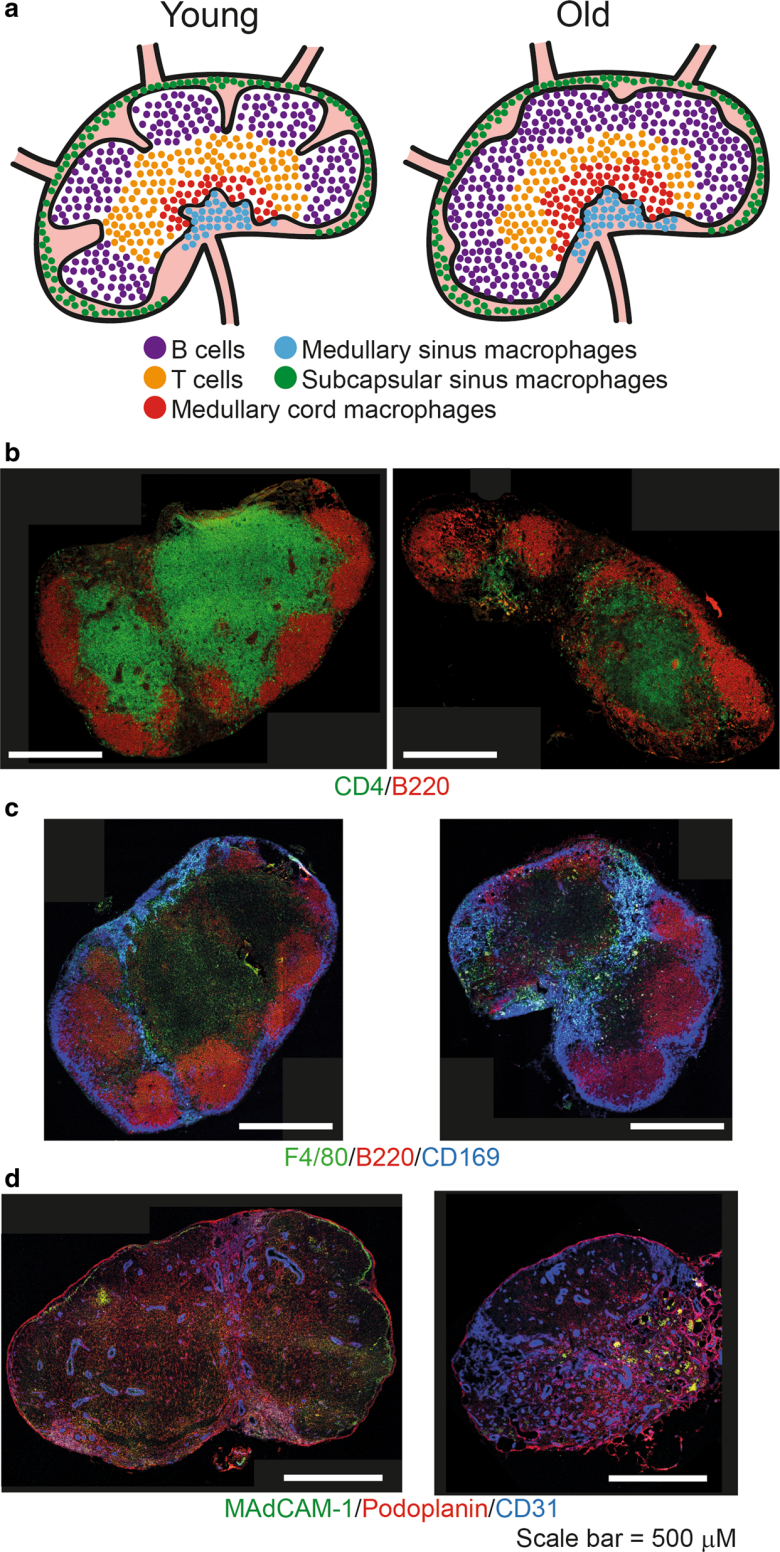
Structural organisation of lymph nodes and changes with age. **a** In a young mouse subcapsular sinus macrophages are located underneath the capsule. On the afferent side of the lymph nodes B cell follicles form distinct follicles in the cortex. Adjacent to the follicle are the T cells in the paracortical area. In the medullary region medullary cord and medullary sinus macrophages are found. In aged lymph nodes B cell follicles no longer form distinct follicles and there is an increase in macrophage populations, especially in the medullary region. **b** Immunofluorescent staining shows changes to lymph nodes with age. Aged lymph nodes no longer form distinct B cell follicles (B220^+^) and there are less T cells. **c** There are increased amounts of subcapsular sinus macrophages (CD169^+^, F4/80^−^), medullary sinus macrophages (CD169^+^, F4/80^+^) and medullary cord macrophages (CD169^−^, F4/80^+^) in aged lymph nodes. **d** Stromal cells are also disrupted with age. An increase in blood endothelial cells (CD31^+^) which form high endothelial venules is evident and are more widely spread throughout the lymph node. Images of inguinal lymph nodes from 2 and 18 month old C57BL/6 mice were acquired by confocal microscopy. Features of this figure are reproduced from (Turner and Mabbott 2017b) under the terms of the CC-BY-NC Creative Commons Attribution Licence 4.0 (Turner and Mabbott 2017b)

The lymph node contains numerous B cell follicles which are distributed throughout the cortex region around the edge of the node. The T cells are localised adjacent to the B cell follicles in the paracortical area (Fig. 3a, b). FDC are found within B cell follicles and support the B cell populations whereas FRC are found in the T cell region to support their functioning (Bajenoff et al. 2006; Willard-Mack 2006). Immune responses then continue in a similar manner to the spleen with germinal centres being formed at the T-B border. Stromal cells such as blood endothelial cells (which form high endothelial venules) and lymphatic endothelial cells help coordinate the movement of cells into, and out of, the blood and lymphatic fluid, respectively (Rouse et al. 1984).

There are three main types of macrophages in the lymph node (Fig. 3a, c). Medullary sinus and medullary cord macrophages are situated in the medullary region and are important in the clearance of pathogens and antigens from the lymph. Subcapsular sinus macrophages, in contrast, are present in a thin layer surrounding the node under the capsule and are adjacent to B cell follicles. Lymphatic fluid enters from the afferent side of the lymph node into the subcapsular sinus region. Thus, these macrophages are the first cells in the lymph node that are exposed to the lymphatic fluid (Gray and Cyster 2012). Subcapsular sinus macrophages are a distinct, poorly endocytic and degradative macrophage subset (Phan et al. 2009). These unique cells capture antigen-containing immune complexes arriving in the lymph node via cell processes that they extend into the lumen of the subcapsular sinus (Junt et al. 2007; Carrasco and Batista 2007; Roozendaal et al. 2009; Phan et al. 2007, 2009). In contrast to other macrophage subsets, subcapsular sinus macrophages retain immune complexes on their surfaces to facilitate their rapid translocation through the floor of the subcapsular sinus to underlying, non-cognate (non-specific) follicular B cells (Phan et al. 2009). These B cells then acquire the immune complexes via their complement receptors and deliver them to FDC. The higher immune complex-binding affinities of FDC most likely strips the B cells of their cargo. Thus, the immune complex relay from subcapsular sinus macrophages to B cells represents an efficient route through which antigens are delivered to FDC (Junt et al. 2007; Carrasco and Batista 2007; Roozendaal et al. 2009; Phan et al. 2007, 2009).

### Changes to lymph node structure and function with age

Studies on tissues from rats, cattle and dogs have together reported no consistent effect of ageing on lymph node weight (Ahmadi et al. 2013). Our own histological analysis of murine lymph nodes has demonstrated altered follicular structure in those from aged mice and disturbances to the delineation of the T cell and B cell zones (Fig. 3b) (Turner and Mabbott 2017b). B cells from aged lymph nodes, in contrast, do not appear to be impaired in their ability to migrate, acquire immune complexes or produce immunoglobulins (Richner et al. 2015; Turner and Mabbott 2017b). The numbers of subcapsular sinus, medullary cord and medullary sinus macrophages is also increased in aged lymph nodes (Fig. 3c) (Turner and Mabbott 2017b).

Stromal cells are important in maintaining the structural integrity of the lymph node and in immune responses. We have observed increased densities of CD31^+^ blood endothelial cells in the aged lymph node (Fig. 3d) (Turner and Mabbott 2017b). These cells are important components of high endothelial venules, but the functional implications of this age-related increase is uncertain. Currently, there are some lymph node stromal cell populations which remain poorly defined and require further characterisation (Malhotra et al. 2012). These undefined cells express many chemokines and cytokines important in immune responses, but whether these cells are also significantly affected by ageing remains to be determined. Mesenteric lymphatic vessels from aged rats also display hyper-permeability due to reductions in the composition of their glycocalyx and GAP junctions (Zolla et al. 2015).

**Fig. 4:**
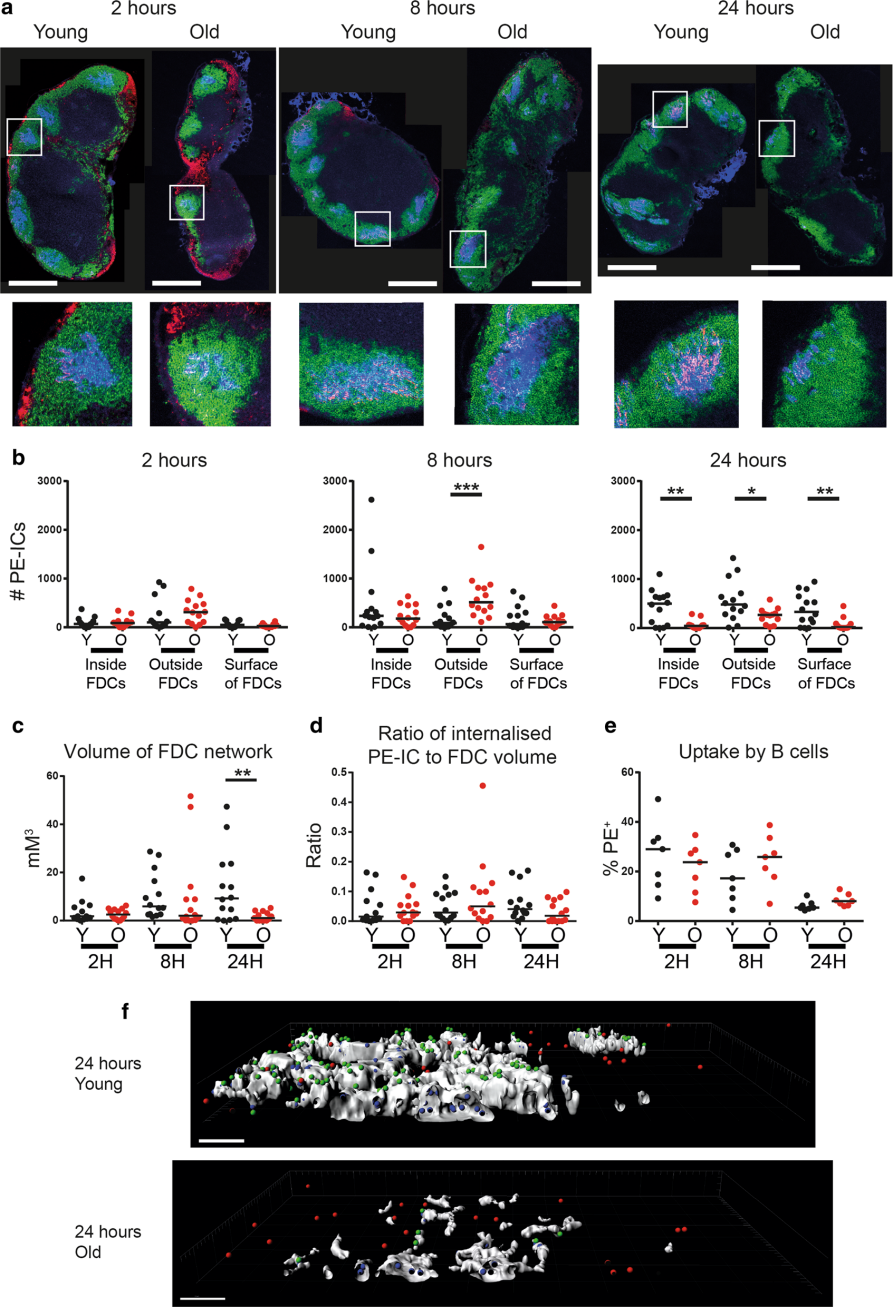
Decreased FDC size impairs immune complex uptake. Young and aged mice were given polyclonal anti-phycoerythrin intraperitoneally 16 h before phycoerytherin was given subcutaneously into each side of the flank, as adapted from a previously published protocol (Phan et al. 2007). Mice were culled 2, 8 and 24 h after phycoerytherin injection and inguinal LN collected for immunostaining. This allowed tracking of phycoerytherin immune complexes (PE-ICs) from their uptake by SCSM, their transference onto B cells, and through to deposition onto FDCs. **a** Immunofluorescent staining of LN from young and aged mice 2, 8 and 24 h post-injection displays PE-ICs (*red*), B cells (B220^+^ *green*) and FDCs (CD35^+^ *blue*) and shows decreased PE-IC localisation to the FDC region at 24 h. Scale bars represent 500 µM. **b** There is a decrease number of PE-ICs inside, on the surface and more than 1 µM away from the surface (*outside*) of FDCs in aged lymph nodes when compared to young lymph nodes at 24 h, as determined via Imaris analysis of z-stack immunostaining. **c** Total volume of FDC networks in the z-stacks imaged at the time points indicated in young and aged mice as determined by Imaris analysis. **d** The ratio of PE-IC complexes inside the FDCs to the FDC volume at the time points indicated shows no difference between young and aged mice. This demonstrates that the decreased FDC volume in aged lymph nodes impedes PE-IC uptake. **e** There was no difference in the uptake of immune complexes between young and old B cells at the time points studied, as determined via flow cytometry. **f** Cross-section representative images of z-stacks post-imaris analysis are shown for young and old mice at 24 h. FDC networks are shown in white. PE-IC complexes inside the FDC network are in *blue*, outside the FDC network in *red* and on the surface of FDC in *green*. *Scale bars* represent 10 µM. Results were analysed via Mann–Whitney *U* test and are pooled from two separate experiments, totalling seven mice per group. *P* < 0 0.001 = ***, *P* < 0.01 = **, *P* < 0.05 = *. Features of this figure are reproduced from (Turner and Mabbott 2017b) under the terms of the CC-BY-NC Creative Commons Attribution Licence 4.0 (Turner and Mabbott 2017b)

FDC are significantly decreased in both their size and number in aged lymph nodes (Szakal et al. 2002, 1988, 1990; Turner and Mabbott 2017b). The ability of FDC to retain immune complexes is also dramatically impaired in the draining lymph nodes of aged mice when compared to those from young mice (Fig. 4a) (Turner and Mabbott 2017b). The decreased size and abundance of FDC in the aged lymph node appears to have a negative impact on germinal centre responses (Aydar et al. 2003; El Shikh et al. 2010; Kosco et al. 1989; Szakal et al. 1988, 1990). Enhanced vulnerability of aged mice to West Nile virus infection has also been proposed to be linked to impaired IgM and IgG responses and delayed germinal centre responses in the draining lymph nodes (Richner et al. 2015).

Impairments in the movement, localisation and response of T cells in the aged lymph node have also been reported (Becklund et al. 2016; Lefebvre et al. 2012; Richner et al. 2015). For example, the recruitment of donor naïve CD4^+^ cells to aged lymph nodes is reduced and coincided with decreased expression of the T cell chemoattractant CCL21 (Richner et al. 2015). The aged lymph node microenvironment has also been shown to decrease naïve T cell survival and homeostatic proliferation (Becklund et al. 2016). Although the maintenance of naïve T cells in the periphery is dependent upon stimulation via the cytokine IL-7, its expression in aged mice and humans was not adversely affected. Instead, ageing effects on stromal cells such as FRC appear to limit the recruitment of naïve T cells to secondary lymphoid tissues and their access to key survival factors (Lefebvre et al. 2012; Becklund et al. 2016).

### Influence of ageing on the immune complex relay from subcapsular sinus macrophages to B cells and the retention of immune complexes by FDC

We have compared the distribution of immune complexes within young and aged murine lymph nodes, from their arrival in the subcapsular sinus to their uptake by FDC. By injecting mice with PE-labelled immune complexes (PE-IC) we were able to observe their initial uptake by subcapsular sinus macrophages, subsequent association with follicular B cells and deposition onto FDC (Fig. 4). At 2 h post-injection, the immune complexes were co-localised with the subcapsular sinus macrophages in draining lymph nodes from each mouse group, but more were present within the inter-follicular regions of aged mice (Fig. 4a) most likely due to the increased abundance of subcapsular sinus macrophages (Fig. 3a). Thus, our data suggested that aged mice displayed no initial impairment in the ability of subcapsular sinus macrophages to take up immune complexes. Furthermore, flow cytometric analysis revealed that there was no significant difference in the ability of young or aged B cells to acquire immune complexes, indicating that the ability of B cells to acquire immune complexes also was not affected by ageing (Fig. 4e) (Turner and Mabbott 2017b).

Whereas large accumulations of immune complexes were detected in association with young FDC by 24 h post-injection, limited amounts were detected in association with aged FDC at this time (Fig. 4a). We also used high-resolution morphometric analysis to compare the relative abundance of immune complexes inside, on the surface and ‘outside’ FDC (>1 µM from the surface; Fig. 4b) (Turner and Mabbott 2017b). Whilst no significant changes were observed at 2 h post-injection in the abundance and distribution of the immune complexes retained by FDC of each age group, by 8 h post-injection more were present outside the aged FDC than those from young mice. At 24 h aged FDC had significantly less immune complexes inside, outside, or on their surfaces (Fig. 4b). Although the total volume of the aged FDC was also reduced when compared to young FDC (Fig. 4c), the immune complex to FDC volume ratio was similar in lymph nodes from young and aged mice (Fig. 4d). This suggests that the impaired ability of FDC to acquire and retain immune complexes in the lymph nodes of aged mice is a consequence of the decreased size of their FDC.

Together our data suggest that the subcapsular sinus macrophage-B cell immune complex relay is unaffected in the draining lymph nodes of aged mice, but the decreased size of aged FDC adversely affects their ability to retain immune complexes (Turner and Mabbott 2017b).

### Human lymph node structure and changes with age

 Human and murine lymph nodes are more similar in structure than their spleens. Human lymph nodes have similar localisation of B cell follicles in the cortex with T cells situated in the adjacent paracortical region (Willard-Mack 2006). Detailed phenotyping of human lymph node macrophages is not as developed as in the mouse, although similar populations are considered to be present (Martens et al. 2006; Angel et al. 2009). Furthermore, research has indicated two distinct lymphatic endothelial cell populations in the human lymph node (Park et al. 2014). The number of lymph nodes reduces with age in humans although no change in the actual size of the lymph node has been reported (Ahmadi et al. 2013). Significant structural changes have been described within ageing human lymph nodes. The area and volume of paracortical, cortex and medullary regions gradually reduces with age (Luscieti et al. 1980) and the medullary cord thins (Ahmadi et al. 2013), supporting the suggestion of an age-related decline in the T cell system. However, the involution of these predominant T cell regions was not observed in all lymph nodes suggestion evidence of some regional variability (Luscieti et al. 1980). Human lymph nodes display a pronounced depletion in naïve T cells, similar to that observed in mice (Lazuardi et al. 2005), most likely a consequence of thymic involution combined with the differentiation of naïve T cells into antigen-specific memory and effector cells. An ageing-associated reduction in CD8^+^ T cells was also observed, but the relative number of CD4^+^ T cells appears to be unaffected by ageing (Lazuardi et al. 2005). A similar decrease in the CD4:CD8 T cell ratio has been observed in the lymph nodes of aged mice (Kirshmann and Murasko 1992). Young individuals with clonal T cell populations in their lymph nodes had increased numbers of CD20^+^ B cells, increased germinal centre size and increased numbers of IgM-expressing cells. Old individuals with clonal T cell populations, in contrast, had lower levels of CD20^+^ B cells and a static follicular microarchitecture (Lazuardi et al. 2005). Indeed, whereas the size of the B cell follicles and B cell abundance was shown to be similar in young and old individuals (Lazuardi et al. 2005), ageing has been associated with absent, or decreased, germinal centre formation (Luscieti et al. 1980). Whether the effects on germinal centres are due to the dysregulated T-B cell interactions within ageing human lymph nodes remains to be determined (Lazuardi et al. 2005; Ahmadi et al. 2013). There is also a documented reduction in the abundance of high endothelial venules (Hadamitzky et al. 2010). On face value, many of these ageing-related changes appear similar to those observed in murine lymph nodes. This suggests that aged murine lymph nodes may represent a physiologically-relevant model in which to further investigate the impact of ageing-related changes to lymph node microarchitecture to the function of the immune cells within.

## Concluding remarks

 The UK elderly population continues to expand due to a combination of factors such as increasing life expectancy and reduced birth rates. A House of Commons report in 2010 estimated that the number of people over 65 years old was predicted to rise to 15.5 million by 2030, and 19 million by 2050 (Cracknell 2010). An independent study of 35 industrialised nations also predicted that there was a >50% probability that female life expectancy in those countries may exceed 90 years old by 2030 (Kontis et al. 2017). The elderly have a decreased response to vaccination and an increased susceptibility to infectious diseases. If significant advances to improved well-being in the human ageing population are not achieved, this significant demographic change has the potential to bring with it important health care system challenges. As reviewed here, large structural changes occur to both spleen and lymph nodes with age which may have significant impact on the functional ability of the immune cells within them. Perhaps if the structural organisation of these organs could be repaired the functioning of the immune system could be boosted. Further cellular and molecular analysis into the effects of ageing on the microarchitecture of secondary lymphoid tissues may therefore help identify therapeutic candidates which could be used to repair or restore the ageing-related disturbances to those tissues, and in doing so, enhance immunity and vaccine responsiveness in the elderly. Despite some important progress in our understanding of how ageing influences the microarchitecture of the spleen and lymph nodes, significant knowledge gaps remain. Much progress has been made from the comparative analysis of ageing rodents, especially mice. But as described above, background strain differences may potentially play a role. Furthermore, there is still much debate concerning whether a species with a substantially reduced lifespan (~2 to 2.5 years) is appropriate to study elderly humans >65 years old. Is an 18–24 months old mouse equivalent to a 70 year old human?
